# Bis(*N*-phenyl­ethane-1,2-diamine)dithio­cyanato­nickel(II)

**DOI:** 10.1107/S1600536809055792

**Published:** 2010-01-09

**Authors:** Chen-Yi Wang, Feng Cao, Ping Wang, Chun-Yan Lv, Xiang Wu

**Affiliations:** a Department of Chemistry, Huzhou University, Huzhou 313000, People’s Republic of China

## Abstract

The asymmetric unit of the title mononuclear Ni^II^ compound, [Ni(NCS)_2_(C_8_H_12_N_2_)_2_], contains two independent half-mol­ecules, the Ni atoms of which lie on crystallographic inversion centres. Each Ni^II^ ion is chelated by two N atoms from two *N*-phenyl­ethane-1,2-diamine ligands and is also coordinated by two N atoms from two thio­cyanate ligands, giving a distorted octa­hedral geometry. In the crystal, mol­ecules are linked into a two-dimensional network parallel to (100) by N—H⋯S inter­actions.

## Related literature

For related structures, see: Lever *et al.* (1983 ▶); Brown & Lingafelter (1963 ▶); Sanni *et al.* (1987 ▶).
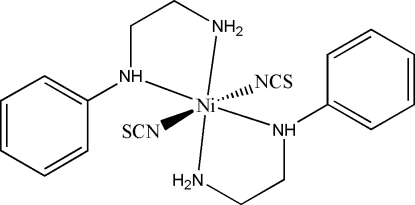


## Experimental

### 

#### Crystal data

[Ni(NCS)_2_(C_8_H_12_N_2_)_2_]
*M* *_r_* = 447.26
Triclinic, 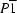

*a* = 7.9947 (2) Å
*b* = 9.4708 (3) Å
*c* = 13.8044 (3) Å
α = 93.045 (1)°
β = 98.258 (1)°
γ = 90.934 (1)°
*V* = 1032.62 (5) Å^3^
*Z* = 2
Mo *K*α radiation
μ = 1.16 mm^−1^
*T* = 298 K
0.18 × 0.17 × 0.17 mm

#### Data collection

Bruker SMART CCD area-detector diffractometer
Absorption correction: multi-scan (*SADABS*; Sheldrick, 1996 ▶) *T*_min_ = 0.819, *T*_max_ = 0.828
6197 measured reflections
4314 independent reflections
3284 reflections with *I* > 2σ(*I*)
*R*_int_ = 0.018

#### Refinement

*R*[*F* ^2^ > 2σ(*F* ^2^)] = 0.037
*wR*(*F* ^2^) = 0.080
*S* = 1.03
4314 reflections
253 parameters
2 restraints
H atoms treated by a mixture of independent and constrained refinement
Δρ_max_ = 0.31 e Å^−3^
Δρ_min_ = −0.26 e Å^−3^

### 

Data collection: *SMART* (Bruker, 1998 ▶); cell refinement: *SAINT* (Bruker, 1998 ▶); data reduction: *SAINT*; program(s) used to solve structure: *SHELXS97* (Sheldrick, 2008 ▶); program(s) used to refine structure: *SHELXL97* (Sheldrick, 2008 ▶); molecular graphics: *SHELXTL* (Sheldrick, 2008 ▶); software used to prepare material for publication: *SHELXTL*.

## Supplementary Material

Crystal structure: contains datablocks global, I. DOI: 10.1107/S1600536809055792/ci5008sup1.cif

Structure factors: contains datablocks I. DOI: 10.1107/S1600536809055792/ci5008Isup2.hkl

Additional supplementary materials:  crystallographic information; 3D view; checkCIF report

## Figures and Tables

**Table 1:** Selected bond lengths (Å)

Ni1—N5	2.073 (2)
Ni1—N1	2.094 (2)
Ni1—N2	2.159 (2)
Ni2—N6	2.047 (2)
Ni2—N3	2.104 (2)
Ni2—N4	2.171 (2)

**Table 2:** Hydrogen-bond geometry (Å, °)

*D*—H⋯*A*	*D*—H	H⋯*A*	*D*⋯*A*	*D*—H⋯*A*
N4—H4*A*⋯S1^i^	0.89 (1)	2.52 (1)	3.393 (2)	168 (3)
N2—H2⋯S2^ii^	0.90 (1)	2.67 (2)	3.436 (2)	144 (3)

Symmetry codes: (i) 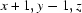; (ii) 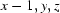.

**Fig. 1.**
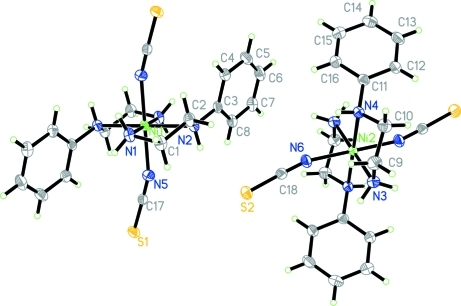
View of the two independent half-molecules in the title compound, showing the atom-numbering scheme. Displacement ellipsoids are drawn at the 30% probability level. Unlabelled atoms in the molecule containing Ni1 are at the symmetry position (-x, 2 - y, -z) and those in the other molecule are at (2 -x, 1 - y, 1 -z).

## supplementary crystallographic information

### Comment

As part of our investigations into novel urease inhibitors, we have synthesized the title compound, a new Ni^II^ complex. There are two independent half-molecules in the asymmetric unit. Each Ni atom lies on an inversion centre and is chelated by two N atoms from two *N*-phenylethane-1,2-diamine ligands, and coordinated by two N atoms from two thiocyanate ligands (Fig. 1). While the three *trans* angles at each Ni centre are 180° by symmetry, the other angles are close to 90° [81.54 (8)°–98.46 (8)°], indicating a slightly distorted octahedral coordination. The Ni—N bond lengths (Table 1) are typical and are comparable with those observed in other similar nickel(II) complexes (Lever *et al.*, 1983; Brown & Lingafelter, 1963; Sanni *et al.*, 1987).

### Experimental

*N*-Phenylethane-1,2-diamine (0.2 mmol, 27.2 mg), ammonium thiocyanate (0.2 mmol, 15.2 mg), and Ni(CH_3_COO)_2_.4H_2_O (0.1 mmol, 24.9 mg) were mixed in a MeOH solution with stirring for 30 min at room temperature. After keeping the filtrate in air for five days, green block-shaped crystals were formed at the bottom of the vessel.

### Refinement

Atoms H2 and H4A were located in a difference Fourier map and refined isotropically, with N-H distances restrained to 0.90 (1) Å. Other H atoms were placed in geometrically idealized positions and constrained to ride on their parent atoms, with C-H distances in the range 0.93–0.97 Å, N–H distances of 0.90 Å, and with *U*_iso_(H) set at 1.2*U*_eq_(C,N).

### Crystal data

**Table:**

[Ni(NCS)_2_(C_8_H_12_N_2_)_2_]	*Z* = 2
*M**_r_* = 447.26	*F*(000) = 468
Triclinic, *P*1	*D*_x_ = 1.438 Mg m^−^^3^
Hall symbol: -P 1	Mo *K*α radiation, λ = 0.71073 Å
*a* = 7.9947 (2) Å	Cell parameters from 1847 reflections
*b* = 9.4708 (3) Å	θ = 2.5–25.0°
*c* = 13.8044 (3) Å	µ = 1.16 mm^−^^1^
α = 93.045 (1)°	*T* = 298 K
β = 98.258 (1)°	Block, green
γ = 90.934 (1)°	0.18 × 0.17 × 0.17 mm
*V* = 1032.62 (5) Å^3^	

### Data collection

**Table:**

Bruker SMART CCD area-detector diffractometer	4314 independent reflections
Radiation source: fine-focus sealed tube	3284 reflections with *I* > 2σ(*I*)
graphite	*R*_int_ = 0.018
ω scan	θ_max_ = 27.0°, θ_min_ = 1.5°
Absorption correction: multi-scan (*SADABS*; Sheldrick, 1996)	*h* = −9→9
*T*_min_ = 0.819, *T*_max_ = 0.828	*k* = −12→8
6197 measured reflections	*l* = −17→17

### Refinement

**Table:**

Refinement on *F*^2^	Primary atom site location: structure-invariant direct methods
Least-squares matrix: full	Secondary atom site location: difference Fourier map
*R*[*F*^2^ > 2σ(*F*^2^)] = 0.037	Hydrogen site location: inferred from neighbouring sites
*wR*(*F*^2^) = 0.080	H atoms treated by a mixture of independent and constrained refinement
*S* = 1.03	*w* = 1/[σ^2^(*F*_o_^2^) + (0.0256*P*)^2^ + 0.3717*P*] where *P* = (*F*_o_^2^ + 2*F*_c_^2^)/3
4314 reflections	(Δ/σ)_max_ = 0.001
253 parameters	Δρ_max_ = 0.31 e Å^−^^3^
2 restraints	Δρ_min_ = −0.26 e Å^−^^3^

### Special details

**Table:**

Geometry. All e.s.d.'s (except the e.s.d. in the dihedral angle between two l.s. planes) are estimated using the full covariance matrix. The cell e.s.d.'s are taken into account individually in the estimation of e.s.d.'s in distances, angles and torsion angles; correlations between e.s.d.'s in cell parameters are only used when they are defined by crystal symmetry. An approximate (isotropic) treatment of cell e.s.d.'s is used for estimating e.s.d.'s involving l.s. planes.
Refinement. Refinement of *F*^2^ against ALL reflections. The weighted *R*-factor *wR* and goodness of fit *S* are based on *F*^2^, conventional *R*-factors *R* are based on *F*, with *F* set to zero for negative *F*^2^. The threshold expression of *F*^2^ > σ(*F*^2^) is used only for calculating *R*-factors(gt) *etc*. and is not relevant to the choice of reflections for refinement. *R*-factors based on *F*^2^ are statistically about twice as large as those based on *F*, and *R*- factors based on ALL data will be even larger.

### Fractional atomic coordinates and isotropic or equivalent isotropic displacement parameters (Å^2^)

**Table:**

	*x*	*y*	*z*	*U*_iso_*/*U*_eq_	
Ni1	0.0000	1.0000	0.0000	0.03175 (12)	
Ni2	1.0000	0.5000	0.5000	0.03243 (12)	
S1	0.21085 (10)	1.40198 (7)	0.20456 (5)	0.0487 (2)	
S2	1.28479 (10)	0.83224 (9)	0.32626 (6)	0.0600 (2)	
N1	0.2337 (3)	0.9430 (2)	−0.04011 (16)	0.0424 (5)	
H1A	0.2188	0.9022	−0.1012	0.051*	
H1B	0.3009	1.0204	−0.0392	0.051*	
N2	0.0504 (3)	0.8295 (2)	0.09707 (15)	0.0348 (5)	
N3	1.2463 (3)	0.4484 (2)	0.56125 (16)	0.0444 (6)	
H3A	1.3139	0.5263	0.5682	0.053*	
H3B	1.2448	0.4147	0.6209	0.053*	
N4	1.0325 (3)	0.3174 (2)	0.40317 (16)	0.0372 (5)	
N5	0.1256 (3)	1.1389 (2)	0.10846 (17)	0.0460 (6)	
N6	1.1013 (3)	0.6271 (2)	0.40688 (16)	0.0435 (6)	
C1	0.3124 (3)	0.8433 (3)	0.0301 (2)	0.0452 (7)	
H1C	0.3664	0.8947	0.0893	0.054*	
H1D	0.3979	0.7902	0.0017	0.054*	
C2	0.1773 (3)	0.7435 (3)	0.0544 (2)	0.0411 (6)	
H2A	0.1247	0.6904	−0.0044	0.049*	
H2B	0.2265	0.6772	0.1010	0.049*	
C3	−0.0938 (3)	0.7571 (2)	0.12332 (18)	0.0345 (6)	
C4	−0.1649 (3)	0.6357 (3)	0.0722 (2)	0.0419 (6)	
H4	−0.1159	0.5953	0.0205	0.050*	
C5	−0.3091 (4)	0.5748 (3)	0.0985 (2)	0.0514 (8)	
H5	−0.3563	0.4933	0.0640	0.062*	
C6	−0.3842 (4)	0.6325 (3)	0.1746 (2)	0.0541 (8)	
H6	−0.4811	0.5906	0.1916	0.065*	
C7	−0.3129 (4)	0.7538 (3)	0.2254 (2)	0.0498 (7)	
H7	−0.3627	0.7941	0.2768	0.060*	
C8	−0.1695 (3)	0.8152 (3)	0.2006 (2)	0.0427 (6)	
H8	−0.1224	0.8963	0.2356	0.051*	
C9	1.3121 (3)	0.3404 (3)	0.4961 (2)	0.0501 (7)	
H9A	1.4042	0.2912	0.5325	0.060*	
H9B	1.3546	0.3851	0.4426	0.060*	
C10	1.1697 (3)	0.2371 (3)	0.4563 (2)	0.0486 (7)	
H10A	1.2089	0.1657	0.4124	0.058*	
H10B	1.1293	0.1902	0.5096	0.058*	
C11	0.8788 (3)	0.2437 (3)	0.36351 (19)	0.0369 (6)	
C12	0.8105 (4)	0.1359 (3)	0.4109 (2)	0.0448 (7)	
H12	0.8716	0.1011	0.4667	0.054*	
C13	0.6509 (4)	0.0801 (3)	0.3749 (2)	0.0532 (8)	
H13	0.6059	0.0080	0.4071	0.064*	
C14	0.5582 (4)	0.1295 (3)	0.2925 (2)	0.0551 (8)	
H14	0.4505	0.0923	0.2697	0.066*	
C15	0.6271 (4)	0.2353 (3)	0.2438 (2)	0.0492 (7)	
H15	0.5663	0.2686	0.1874	0.059*	
C16	0.7864 (3)	0.2914 (3)	0.2791 (2)	0.0428 (6)	
H16	0.8321	0.3621	0.2459	0.051*	
C17	0.1622 (3)	1.2472 (3)	0.14957 (18)	0.0353 (6)	
C18	1.1772 (3)	0.7109 (3)	0.37193 (18)	0.0372 (6)	
H2	0.104 (4)	0.874 (3)	0.1524 (14)	0.080*	
H4A	1.076 (4)	0.353 (3)	0.3537 (17)	0.080*	

### Atomic displacement parameters (Å^2^)

**Table:**

	*U*^11^	*U*^22^	*U*^33^	*U*^12^	*U*^13^	*U*^23^
Ni1	0.0354 (3)	0.0256 (2)	0.0348 (3)	−0.00085 (18)	0.00618 (19)	0.00362 (18)
Ni2	0.0350 (3)	0.0293 (2)	0.0333 (3)	−0.00465 (19)	0.00483 (19)	0.00667 (19)
S1	0.0621 (5)	0.0367 (4)	0.0477 (4)	−0.0126 (3)	0.0130 (4)	−0.0036 (3)
S2	0.0544 (5)	0.0756 (6)	0.0502 (5)	−0.0305 (4)	0.0059 (4)	0.0199 (4)
N1	0.0442 (13)	0.0376 (12)	0.0484 (14)	0.0029 (10)	0.0135 (11)	0.0114 (10)
N2	0.0373 (12)	0.0311 (11)	0.0371 (13)	−0.0011 (9)	0.0079 (10)	0.0054 (9)
N3	0.0426 (13)	0.0445 (13)	0.0439 (14)	−0.0035 (10)	−0.0003 (10)	0.0028 (11)
N4	0.0372 (12)	0.0370 (12)	0.0374 (13)	−0.0042 (10)	0.0050 (10)	0.0053 (10)
N5	0.0558 (15)	0.0365 (13)	0.0436 (14)	−0.0056 (11)	0.0019 (11)	0.0002 (11)
N6	0.0518 (15)	0.0391 (13)	0.0415 (13)	−0.0066 (11)	0.0126 (11)	0.0075 (10)
C1	0.0382 (16)	0.0445 (16)	0.0552 (18)	0.0092 (12)	0.0096 (13)	0.0145 (13)
C2	0.0434 (16)	0.0325 (14)	0.0494 (17)	0.0092 (12)	0.0090 (13)	0.0120 (12)
C3	0.0353 (14)	0.0312 (13)	0.0375 (14)	0.0001 (11)	0.0026 (11)	0.0132 (11)
C4	0.0494 (17)	0.0345 (14)	0.0419 (16)	0.0006 (12)	0.0049 (13)	0.0080 (12)
C5	0.0481 (18)	0.0373 (16)	0.067 (2)	−0.0073 (13)	−0.0003 (15)	0.0110 (14)
C6	0.0368 (16)	0.0563 (19)	0.071 (2)	−0.0027 (14)	0.0066 (15)	0.0288 (17)
C7	0.0505 (18)	0.0519 (18)	0.0511 (18)	0.0062 (14)	0.0161 (14)	0.0153 (14)
C8	0.0485 (17)	0.0408 (15)	0.0411 (16)	0.0003 (12)	0.0124 (13)	0.0089 (12)
C9	0.0384 (16)	0.0549 (18)	0.0569 (19)	0.0059 (13)	0.0047 (14)	0.0046 (15)
C10	0.0473 (17)	0.0423 (16)	0.0556 (19)	0.0067 (13)	0.0061 (14)	−0.0004 (14)
C11	0.0413 (15)	0.0308 (13)	0.0386 (15)	−0.0011 (11)	0.0084 (12)	−0.0030 (11)
C12	0.0545 (18)	0.0361 (15)	0.0440 (16)	−0.0061 (13)	0.0080 (13)	0.0040 (12)
C13	0.061 (2)	0.0409 (16)	0.059 (2)	−0.0167 (14)	0.0143 (16)	0.0009 (14)
C14	0.0462 (18)	0.0518 (18)	0.065 (2)	−0.0127 (14)	0.0053 (15)	−0.0053 (16)
C15	0.0478 (18)	0.0529 (18)	0.0453 (17)	0.0017 (14)	0.0027 (13)	−0.0008 (14)
C16	0.0440 (16)	0.0426 (15)	0.0422 (16)	−0.0039 (12)	0.0070 (13)	0.0059 (12)
C17	0.0330 (14)	0.0420 (15)	0.0318 (14)	−0.0013 (11)	0.0054 (11)	0.0091 (12)
C18	0.0376 (15)	0.0402 (15)	0.0335 (14)	−0.0013 (12)	0.0048 (11)	0.0014 (11)

### Geometric parameters (Å, °)

**Table:**

Ni1—N5	2.073 (2)	C1—H1D	0.97
Ni1—N5^i^	2.073 (2)	C2—H2A	0.97
Ni1—N1^i^	2.094 (2)	C2—H2B	0.97
Ni1—N1	2.094 (2)	C3—C4	1.386 (3)
Ni1—N2^i^	2.159 (2)	C3—C8	1.393 (4)
Ni1—N2	2.159 (2)	C4—C5	1.384 (4)
Ni2—N6^ii^	2.047 (2)	C4—H4	0.93
Ni2—N6	2.047 (2)	C5—C6	1.376 (4)
Ni2—N3^ii^	2.104 (2)	C5—H5	0.93
Ni2—N3	2.104 (2)	C6—C7	1.384 (4)
Ni2—N4^ii^	2.171 (2)	C6—H6	0.93
Ni2—N4	2.171 (2)	C7—C8	1.372 (4)
S1—C17	1.629 (3)	C7—H7	0.93
S2—C18	1.630 (3)	C8—H8	0.93
N1—C1	1.473 (3)	C9—C10	1.511 (4)
N1—H1A	0.90	C9—H9A	0.97
N1—H1B	0.90	C9—H9B	0.97
N2—C3	1.432 (3)	C10—H10A	0.97
N2—C2	1.479 (3)	C10—H10B	0.97
N2—H2	0.898 (10)	C11—C12	1.387 (3)
N3—C9	1.479 (3)	C11—C16	1.388 (4)
N3—H3A	0.90	C12—C13	1.386 (4)
N3—H3B	0.90	C12—H12	0.93
N4—C11	1.426 (3)	C13—C14	1.374 (4)
N4—C10	1.475 (3)	C13—H13	0.93
N4—H4A	0.889 (10)	C14—C15	1.386 (4)
N5—C17	1.158 (3)	C14—H14	0.93
N6—C18	1.158 (3)	C15—C16	1.383 (4)
C1—C2	1.508 (3)	C15—H15	0.93
C1—H1C	0.97	C16—H16	0.93
			
N5—Ni1—N5^i^	180	N1—C1—H1D	109.9
N5—Ni1—N1^i^	90.87 (9)	C2—C1—H1D	109.9
N5^i^—Ni1—N1^i^	89.13 (9)	H1C—C1—H1D	108.3
N5—Ni1—N1	89.13 (9)	N2—C2—C1	107.7 (2)
N5^i^—Ni1—N1	90.87 (9)	N2—C2—H2A	110.2
N1^i^—Ni1—N1	180	C1—C2—H2A	110.2
N5—Ni1—N2^i^	90.77 (8)	N2—C2—H2B	110.2
N5^i^—Ni1—N2^i^	89.23 (8)	C1—C2—H2B	110.2
N1^i^—Ni1—N2^i^	82.40 (8)	H2A—C2—H2B	108.5
N1—Ni1—N2^i^	97.60 (8)	C4—C3—C8	119.0 (2)
N5—Ni1—N2	89.23 (8)	C4—C3—N2	122.6 (2)
N5^i^—Ni1—N2	90.77 (8)	C8—C3—N2	118.3 (2)
N1^i^—Ni1—N2	97.60 (8)	C5—C4—C3	119.6 (3)
N1—Ni1—N2	82.40 (8)	C5—C4—H4	120.2
N2^i^—Ni1—N2	180	C3—C4—H4	120.2
N6^ii^—Ni2—N6	180	C6—C5—C4	121.3 (3)
N6^ii^—Ni2—N3^ii^	89.13 (9)	C6—C5—H5	119.3
N6—Ni2—N3^ii^	90.87 (9)	C4—C5—H5	119.3
N6^ii^—Ni2—N3	90.87 (9)	C5—C6—C7	118.8 (3)
N6—Ni2—N3	89.13 (9)	C5—C6—H6	120.6
N3^ii^—Ni2—N3	180	C7—C6—H6	120.6
N6^ii^—Ni2—N4^ii^	89.54 (8)	C8—C7—C6	120.6 (3)
N6—Ni2—N4^ii^	90.46 (8)	C8—C7—H7	119.7
N3^ii^—Ni2—N4^ii^	81.54 (8)	C6—C7—H7	119.7
N3—Ni2—N4^ii^	98.46 (8)	C7—C8—C3	120.6 (3)
N6^ii^—Ni2—N4	90.46 (8)	C7—C8—H8	119.7
N6—Ni2—N4	89.54 (8)	C3—C8—H8	119.7
N3^ii^—Ni2—N4	98.46 (8)	N3—C9—C10	108.3 (2)
N3—Ni2—N4	81.54 (8)	N3—C9—H9A	110.0
N4^ii^—Ni2—N4	180	C10—C9—H9A	110.0
C1—N1—Ni1	108.38 (16)	N3—C9—H9B	110.0
C1—N1—H1A	110.0	C10—C9—H9B	110.0
Ni1—N1—H1A	110.0	H9A—C9—H9B	108.4
C1—N1—H1B	110.0	N4—C10—C9	107.8 (2)
Ni1—N1—H1B	110.0	N4—C10—H10A	110.1
H1A—N1—H1B	108.4	C9—C10—H10A	110.1
C3—N2—C2	117.70 (19)	N4—C10—H10B	110.1
C3—N2—Ni1	116.59 (15)	C9—C10—H10B	110.1
C2—N2—Ni1	104.74 (15)	H10A—C10—H10B	108.5
C3—N2—H2	107 (2)	C12—C11—C16	118.8 (2)
C2—N2—H2	107 (2)	C12—C11—N4	122.6 (2)
Ni1—N2—H2	103 (2)	C16—C11—N4	118.2 (2)
C9—N3—Ni2	109.24 (16)	C13—C12—C11	119.9 (3)
C9—N3—H3A	109.8	C13—C12—H12	120.1
Ni2—N3—H3A	109.8	C11—C12—H12	120.1
C9—N3—H3B	109.8	C14—C13—C12	121.2 (3)
Ni2—N3—H3B	109.8	C14—C13—H13	119.4
H3A—N3—H3B	108.3	C12—C13—H13	119.4
C11—N4—C10	118.3 (2)	C13—C14—C15	119.2 (3)
C11—N4—Ni2	114.31 (16)	C13—C14—H14	120.4
C10—N4—Ni2	105.40 (16)	C15—C14—H14	120.4
C11—N4—H4A	108 (2)	C16—C15—C14	120.0 (3)
C10—N4—H4A	105 (2)	C16—C15—H15	120.0
Ni2—N4—H4A	104 (2)	C14—C15—H15	120.0
C17—N5—Ni1	156.7 (2)	C15—C16—C11	120.9 (3)
C18—N6—Ni2	165.9 (2)	C15—C16—H16	119.6
N1—C1—C2	108.8 (2)	C11—C16—H16	119.6
N1—C1—H1C	109.9	N5—C17—S1	178.3 (3)
C2—C1—H1C	109.9	N6—C18—S2	178.0 (2)

Symmetry codes: (i) −*x*, −*y*+2, −*z*; (ii) −*x*+2, −*y*+1, −*z*+1.

### Hydrogen-bond geometry (Å, °)

**Table:**

*D*—H···*A*	*D*—H	H···*A*	*D*···*A*	*D*—H···*A*
N4—H4A···S1^iii^	0.89 (1)	2.52 (1)	3.393 (2)	168 (3)
N2—H2···S2^iv^	0.90 (1)	2.67 (2)	3.436 (2)	144 (3)

Symmetry codes: (iii) *x*+1, *y*−1, *z*; (iv) *x*−1, *y*, *z*.
